# How Identification With the Social Environment and With the Government Guide the Use of the Official COVID-19 Contact Tracing App: Three Quantitative Survey Studies

**DOI:** 10.2196/28146

**Published:** 2021-11-24

**Authors:** Annika Scholl, Kai Sassenberg

**Affiliations:** 1 Social Processes Lab Leibniz-Institut fuer Wissensmedien Tuebingen Germany

**Keywords:** COVID-19, SARS-CoV-2, contact tracing app, social identification, technology acceptance, pandemic, outbreak, health technology

## Abstract

**Background:**

Official contact tracing apps have been implemented and recommended for use across nations to track and contain the spread of COVID-19. Such apps can be effective if people are *willing* to use them. Accordingly, many attempts are being made to motivate citizens to make use of the officially recommended apps.

**Objective:**

The aim of this research was to contribute to an understanding of the preconditions under which people are willing to use a COVID-19 contact tracing app (ie, their use intentions and use). To go beyond personal motives in favor of app use, it is important to take people’s social relationships into account, under the hypothesis that the more people identify with the *beneficiaries* of app use (ie, people living close by in their social environment) and with the *source* recommending the app (ie, members of the government), the more likely they will be to accept the officially recommended contact tracing app.

**Methods:**

Before, right after, and 5 months after the official contact tracing app was launched in Germany, a total of 1044 people participated in three separate surveys. Structural equation modeling was used to test the hypotheses, examining the same model in all studies at these critical points in time.

**Results:**

Across the three surveys, both identification with the beneficiaries (people living in their social environment) and with the source recommending the app (members of the government) predicted greater intention to use and use (installation) of the official contact tracing app. Trust in the source (members of the government) served as a mediator. Other types of identification (with people in Germany or people around the world) did not explain the observed results. The findings were highly consistent across the three surveys.

**Conclusions:**

Attempts to motivate people to use new health technology (or potentially new measures more generally) not only for their personal benefit but also for collective benefits should take the social context into account (ie, the social groups people belong to and identify with). The more important the beneficiaries and the sources of such measures are to people’s sense of the self, the more willing they will likely be to adhere to and support such measures.

## Introduction

### Background

The outbreak of COVID-19 in 2020 impacted individual people worldwide but also their communities, governments, and whole nations, with (often) unknown challenges and numerous new measures to be rapidly accepted and implemented. One important measure implemented in many countries to collectively contain the spread of the virus has been the use of new technological means, namely, an *official contact tracing app* [1]. Such apps aim at retracing chains of infection and warning people in case they have had contact with a potentially ill person. In Germany, the official app was commissioned by the German government—launched on June 16, 2020—and has since been recommended by the government for (voluntary) use. In February 2021, almost 8 months after its launch, 25.4 million people (out of roughly 83.7 million living in Germany) were using the app, with 59% positive test results being entered [2]. Therefore, there is still substantial room to gain more users. This situation raises the question: What *motivates* people to support and make use of such new technology?

In an “era of massive technological advancement” [3] and this pandemic, studying the acceptance of such health-related apps (especially regarding contact tracing) is important both from theoretical and practical points of view to better understand and potentially foster people’s willingness to use them. Going beyond prior work on personal motives for app use, this study examined the role of social relationships. Specifically, we targeted the question if the extent to which people identify with specific groups, namely (a) with the *beneficiaries* of app use (ie, people in their social environment) and (b) with the *source* recommending the app (ie, members of the government), predicts a greater willingness to use the contact tracing app. This relies on the idea that tracing apps gain their impact at the collective level (ie, when many people use them, these apps are beneficial for the community but not necessarily for the individual user).

We used three separate surveys to test this idea at crucial points during 2020: right before the official app was launched, right after its launch, and 5 months later when substantial extensions to track more user data were added. With this approach, we sought to contribute to a better understanding of the preconditions of people’s willingness to use such new health-related technology from a motivational perspective, highlighting the importance of the social groups (collectives) that people belong to (rather than their personal benefits or pitfalls) as driving forces.

### Prior Work on App Acceptance and the Role of Individual Motives as a Predictor

This study focused on the official contact tracing app in Germany. This specific app traces contact of the user with people who have been diagnosed with COVID-19, using Bluetooth over smartphones. If a “positive” contact is recorded during the day, the app notifies the user with a warning the next day, which did not reveal the potential contact’s identity or the specific point in time of contact when the study was performed.

Prior work on how people respond to such apps focused on three main aspects. First, recent reviews compared the features of tracing apps and other types of apps developed during the COVID-19 pandemic (eg, for training, information sharing, or diagnosis) [4-6]. In these reviews, the focus was on the descriptions of the technology rather than the users. Second, researchers have performed surveys on general *acceptance rates* of COVID-19 tracing apps among different populations. Although some findings suggest relatively high support for the app across countries [7], other studies did point out the problem of low usage rates (eg, in France among health care students) [8]. In Germany, at the time of the launch of the official contact tracing app, 81% of an adult sample between 18-77 years old did possess the (technological and ability-related) requirements to use the app, but only 35% reported being willing to do so [9]. This points to the necessity to better understand the *motivational* preconditions of app acceptance.

The low acceptance rates stress the relevance of the third research question on what motivates people to make use of this app and disclose their personal data. In this regard, prior work has adopted a clear focus on *personal* benefits and detriments. From a personal perspective, using these technological means does carry benefits (eg, being informed about one’s own positive contacts) but also potential barriers for each individual user (eg, providing personal data). Personal motives are known to influence use intentions. Recent evidence in this domain has shown that privacy concerns along with uncertainty about the app’s effectiveness constitute two main personal *barriers* to accept such an app [5,7,8,10-12]; thus, balancing these two aspects poses a major challenge for app developers [3,13]. At the same time, personal conditions that *support* acceptance are related to already having adopted one’s lifestyle during the pandemic [14], trusting in data security or authorities [7,10,11], perceiving high personal vulnerability to a health threat [12], and experiencing high personal self-efficacy [12]. Finally, providing users with an app that seems easy to use [15] or giving transparent information about the app contributes to greater app acceptance [11].

Overall, motivational factors on the level of the *individual* play a role in acceptance: people are more willing to use technological means if they expect it to benefit (rather than cost) them personally [16]. However, it is unclear whether such personal costs and benefits are the *only* motivational drivers, or if people might also be motivated to use the app (as a measure designed for a collective) not for personal reasons but because they care about *others*. Going beyond the prior work outlined above, we here present a novel perspective on the motivational drivers behind app acceptance. We reason that people may also be willing to use the app because they *identify* with those who benefit from the app and/or those who recommend using it.

### The Role of Identification With Others (Beneficiaries) in Fostering App Use

One important aspect of technological means such as a contact tracing app during a pandemic is that its use does not only benefit oneself personally but it also benefits *other people* in one’s social environment (ranging from friends or family living close by to unknown people who happen to buy their groceries in the same supermarket) by warning them in case one receives a positive test result. Accordingly, people may be more motivated to use the app the more *important* the welfare of these potential beneficiaries is to them.

Others’ importance to oneself is a topic addressed Social Identity Theory [17,18]. People define themselves not only in terms of what makes them unique individuals (“I”; their personal identity) but also in terms of the social groups they belong to (“we”; their social identity). The more a person identifies with a social group they belong to, the more relevant this (in)group becomes to their definition of the “self.” As a result, people start thinking more in terms of “we” (rather than “I”) and they care more about the interests of the group. Consequently, the more people identify with a social group, the more willing they are to engage in behaviors that benefit the group’s members (eg, use technology to contribute knowledge for others at work) [19-21], potentially even at personal costs (eg, sacrificing privacy concerns to benefit the safety of the group; see the example of CCTV cameras in the United Kingdom [22]).

Applied to the present case, this implies that the more a person identifies with people living in their social environment (ie, potential beneficiaries from their app use), the more willing this person will likely be to contribute to these others’ welfare and thus to use the app. This resulted in the following hypothesis:

*H1: The more people identify with people living in their social environment, the more willing they are to use the contact tracing app.*

### The Role of Identification With the Source in Fostering App Use

A second important motivational predictor of people’s willingness to use such new technology could be the level of identification with the *source* (“authority”) recommending the app. In this sense, the more people trust in and identify with members of the government—as the source who commissioned the production of this app and now persistently recommends its use—the more willing they may be to use the app.

Members of the government represent a small group of (elected) people who act as representatives of a nation (ie, the broader ingroup of the people of their country that they may identify with). In the present case, we investigated people’s identification with the *members of the German government*. During the early months of the COVID-19 pandemic, European and international news often stated that the German government was dealing relatively effectively with the challenges; during these times, members of the government have been meeting and communicating the results of such meetings repeatedly (eg, every other week) to the public to address citizens’ concerns and needs (eg, building upon regular opinion surveys and including public addresses). As a result, citizens may have had the impression of having substantial (virtual) contact with members of the government, and such (positive) contact is known to create a feeling of closeness and identification to others [23,24]. In short, citizens may have had opportunities to *identify* with members of the government (even if citizens would typically not consider them to be members of their ingroup) just as people can generally identify with their leaders at work (eg, [25-28]).

The more people identify with others, the more positively they view these others [29-32]. This also means that when taking a “leap of faith” and being potentially vulnerable (as is likely the case during a pandemic), identification presumably makes people more willing to *trust* in and follow their ingroup members [33,34] and, potentially, even to trust more in and follow those people (authorities) who make decisions on behalf of their ingroup (eg, their community [35] or the people of their country). Supporting this idea, trust in the government was an important predictor of accepting measures during the Ebola outbreak [36,37] (for a similar argument, see [38]), and has also been considered important by people themselves during the COVID-19 pandemic [10]. This resulted in the following hypothesis:

*H2: The more people identify with members of the government, the more they trust in these members and, accordingly, the more they are willing to use the contact tracing app.*

### Study Objectives and Design

These two predictions were tested across three surveys at different important points in time throughout 2020 to perform a more comprehensive test of the hypothesized model. As an indicator of people’s responses toward the contact tracing app, all surveys focused on the outcome willingness to accept the app (*app acceptance*); we additionally assessed willingness to use the app prior to its launch (*intentions to use*) or after its launch (*app use*; reflecting that people had already installed the app on their smartphones).

**Figure 1:**
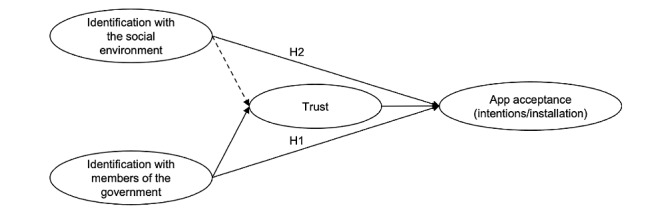
General model tested across the three surveys. The dashed line reflects an exploratory path and the solid lines reflect hypothesized paths. H: hypothesis.

Taken together, we hypothesized that the more people identify (1) with other people in their social environment and/or (2) with members of the government, the greater their willingness to use the official contact tracing app should be. For the latter (H2), trust in the government was the assumed mediator. For the former (H1), we did not predict a mediating role of trust; notwithstanding, we explored whether trust would also serve as a potential mediator for identification with the social environment in predicting greater app acceptance. The general model is presented in Figure 1.

As such, the broader aim of this study was to contribute to a better understanding of the preconditions of people’s willingness to use this new technology from a motivational perspective. We sought to demonstrate, for the first time, the importance of (identification with) the social groups that people belong to as a driver toward greater app acceptance.

## Methods

### Procedure and Sample

At all three time points, participants were invited via a university email to complete a brief (7-10 minutes) survey on their perception of the current (COVID-19–related) situation. Participants received basic information about the respective study (eg, duration, compensation, purpose), provided informed consent, completed the main measures as indicated below, entered demographic information, were debriefed, confirmed their consent (or withdrew it) to use their survey data, and were finally given the chance to take part in a lottery of gift vouchers. Note that Surveys 2 and 3 included an experimental manipulation making identification with the government/environment salient (compared to control groups). This manipulation turned out to be ineffective; we thus analyzed and report results by means of correlations between measured constructs. More information on this aspect is certainly available from the authors. The local ethics committee provided ethical approval for all studies.

From May 25 to May 26, 2020, right before the official app was launched in Germany, 355 participants completed Survey 1 (268 women, 81 men, 6 diverse/nonspecified; mean age 23.53 years, SD 5.827, range 18-80 years); two additional participants of Survey 1 (none in Survey 2 and one in Survey 3) retracted their data after the debriefing, which were accordingly deleted prior to any analysis. Survey 2 (June 16, 2020, right after the app was launched) included 308 nonoverlapping participants (228 women, 74 men, 3 diverse/nonspecified, 3 missing; mean age 23.93 years, SD 5.79, range 18-73 years). Survey 3 (November 23-25, 2020, when additional app functions requiring more personal data were discussed) involved another separate sample of 381 participants (278 women, 100 men, 3 diverse/nonspecified; mean age 22.57 years, SD 4.83, range 18-69 years).

### Measures

#### Source Data and Code

The exact order of measures in each study (including potential additional control measures) and the original materials for all surveys are reported in Multimedia Appendix 1 and Multimedia Appendix 2. Data [39] and code [40] are available at PsychArchives.

#### Identification

Identification with (1) people in their social environment and (2) members of the government, as our main predictors, were measured with six items each, adapted from McFarland et al [41] (eg, “How connected do you feel to the following groups?”: social environment (1=*nothing* to 7=*very/very much*).

As a control variable, we assessed identification with two broader (in)groups that people may identify with to rule out that the relations we predict are driven by these aspects of identification. In Surveys 1 and 2, we assessed (with the same items) (3) identification with *people living in Germany* as a control variable, whereas in Survey 3, we assessed (4) identification with *people around the world* (humanity) as a control.

For exploratory purposes, we also assessed identification with *scientists in the health domain* as potential “users” of the app data; however, as this measure was strongly correlated with *identification with members of the government* (Survey 1, N=355: *r*=0.542, *P*<.001; Survey 2, N=308*: r*=0.473, *P*<.001), we refrained from further analyses with this measure.

#### Trust

Trust in the government as a mediator was measured with four items in all surveys to assess the trust dimensions as indicated by Mayer et al [42], adapted from Winter et al [43] (eg, “How trustworthy/honest/competent/credible do you perceive the government to be?”: 1=*not at all* to 7=*very*).

#### App Acceptance

App acceptance as a first outcome was operationalized as low perceived privacy infringement. Participants in Survey 1 (prior to launch of the app) received a brief description that the government was currently *planning on launching* the app for voluntary use to limit the spread of the virus, potentially trace users’ movements, and warn users in case of a high-risk contact; in Survey 2 (after the launch), this message stated that the government had just initiated the launch of the app (by June 16, with the same purpose as indicated for Survey 1). Participants in Surveys 1 and 2 indicated how they perceived the call to use such an app with six items, adapted from Alge et al [44] (eg, “I find it acceptable that such an app should be used”: 1=*does not apply at all* to 7=*totally applies*).

In Survey 3, we assessed this outcome 5 months (November 2020) after the app had been launched and an extension by further functions was addressed to collect more extensive user data as a basis for new governmental measures. Accordingly, this outcome was operationalized in terms of people’s acceptance of *more app functions* (meaning providing more personal data) with 8 items adapted from Surveys 1 and 2 (eg, “I would be willing to disclose more information from myself as a basis to decide on new measures”: 1=*does not apply at all* to 7=*totally applies*).

#### Intention to Use the App and Use (Installation) of the App

The *intention* to use the app as a second outcome in Surveys 1 and 2 was assessed with one item: “To which extent would you be/are you willing to use this contact-tracing app?” (1=*not at all* to 7=*very much*). The *use* of the contact tracing app was assessed as an additional outcome in Survey 2; we operationalized use by asking participants whether they had already installed the official or another contact tracing app (0=no, 1=yes: 25.3% stating yes; mean 0.25, SD 0.44). To be better able to differentiate whether agreement in this case referred to the official (ie, government-recommended) app, in Survey 3, we specifically assessed the use of this official app with the question: “Is the official contact tracing app installed on your smartphone?” (0=no, 1=yes, missing=unsure/different app: 57.7% stating yes, 41.2% no, and 1% unsure; mean 0.58, SD 0.49). As such, use was operationalized as *installation of the app* (ie, having installed the tracing app on their smartphones).

### Data Analysis

We analyzed the data using structural equation modeling with Mplus 8.4 [45]. The tested model for each survey examined the relationship between the predictors (identification with the government, identification with the social environment), controlling for identification with people in Germany (Surveys 1-2) or for identification with humanity (Survey 3); the mediator trust; and the outcomes app acceptance (Surveys 1-3), intention to use the app (Surveys 1-2), and either the use of some contact tracing app (Survey 2) or the use of the official contact tracing app (Survey 3). Moreover, we tested for *indirect effects* of identification (with the government, with the social environment) in predicting more app acceptance, intention to use the app, and/or use of the app via greater trust. We hypothesized and tested an indirect effect of identification with the government via trust on the outcomes (H1); in addition, we explored if trust would also serve as a “linking mechanism” between identification with the social environment and the outcomes (ie, also for the prediction in H2).

## Results

### Descriptive Statistics

Descriptive statistics and Cronbach α values are shown in Table 1. The intercorrelations among measures for each study are reported in Tables 2-4. Items were originally in German and translated for this paper.

**Table 1:** Descriptive statistics and bivariate correlations for the three surveys.

Variable	Survey 1 (N=355)			Survey 2 (N=308)			Survey 3 (N=381)	
	Mean (SD)	Cronbach α	Mean (SD)		Cronbach α	Mean (SD)		Cronbach α
Identification with the social environment	6.29 (0.79)	.89	6.24 (0.83)		.91	6.38 (0.63)		.88
Identification with members of the government	3.11 (1.15)	.87	3.32 (1.19)		.91	3.01 (1.16)		.88
Identification with people in Germany^a^ (control)	4.52 (1.03)	.87	4.57 (1.01)		.85	4.54 (1.11)		.90
Trust	4.35 (1.32)	.92	4.68 (1.28)		.93	4.73 (1.19)		.91
App acceptance^b^	4.33 (1.56)	.91	5.01 (1.41)		.89	4.44 (1.62)		.94
Intention to use the app (1 item)	4.50 (1.89)	N/A^c^	4.29 (2.14)		N/A	N/A		N/A
Installation of the app^d^	N/A	N/A	0.25 (0.44)		N/A	0.58 (0.49)		N/A

^a^For Survey 3, this control variable was identification with people around the world.

^b^Referred to more app functions for Survey 3.

^c^N/A: not applicable.

^d^0=no, 1=yes for Surveys 1-2; 0=no, 1=yes, unsure=missing for Survey 3.

**Table 2:** Correlation analysis (Pearson *r* and two-tailed *P* value) of study variables in Survey 1 (N=355).

Variable		Identification with social environment	Identification with government	Identification with people in Germany (control)	Trust	App acceptance	Intention to use app (1 item)
**Identification with social environment**							
	*r*	1	0.21	0.35	0.26	0.08	0.11
	*P* value	—^a^	<.001	<.001	<.001	.15	.045
**Identification with government**							
	*r*	0.21	1	0.47	0.53	0.22	0.19
	*P* value	<.001	—	<.001	<.001	<.001	<.001
**Identification with people in Germany (control)**							
	*r*	0.35	0.47	1	0.28	0.08	0.08
	*P* value	<.001	<.001	—	<.001	.15	.13
**Trust**							
	*r*	0.26	0.53	0.28	1	0.50	0.49
	*P* value	<.001	<.001	<.001	—	<.001	<.001
**App acceptance**							
	*r*	0.08	0.22	0.08	0.49	1	0.81
	*P* value	.15	<.001	.15	<.001	—	<.001
**Intention to use app (1 item)**							
	*r*	0.11	0.19	0.08	0.49	0.81	1
	*P* value	0.045	<.001	.13	<.001	<.001	—

^a^Not applicable.

**Table 3:** Correlation analysis (Pearson *r* and two-tailed *P* value) among variables in Survey 2 (N=308).

Variable		Identification with social environment	Identification with government	Identification with people in Germany (control)	Trust	App acceptance	Intention to use app (1 item)	Installation of app (0=no, 1=yes/no)	
**Identification with social environment**									
	*r*	1	0.26	0.46	0.26	0.07	0.11	–0.02	
	*P* value	—^a^	<.001	<.001	<.001	.22	.05	.76	
**Identification with government**									
	*r*	0.26	1	0.57	0.52	0.24	0.25	0.16	
	*P* value	<.001	—	<.001	<.001	<.001	<.001	.004	
**Identification with people in Germany (control)**									
	*r*	0.46	0.57	1	0.32	0.02	0.03	0.03	
	*P* value	<.001	<.001	—	<.001	.72	.62	.62	
**Trust**									
	*r*	0.26	0.52	0.32	1	0.47	0.46	0.23	
	*P* value	<.001	<.001	<.001	—	<.001	<.001	<.001	
**App acceptance**									
	*r*	0.07	0.24	0.02	0.47	1	0.78	0.51	
	*P* value	.22	<.001	.72	<.001	—	<.001	<.001	
**Intention to use app (1 item)**									
	*r*	0.11	0.25	0.03	0.46	0.78	1	0.54	
	*P* value	.05	<.001	.62	<.001	<.001	—	<.001	
**Installation of app (0=no, 1=yes/no)**									
	*r*	–0.02	0.16	0.04	0.23	0.51	0.54	1	
	*P* value	.76	.004	.45	<.001	<.001	<.001	—	

^a^Not applicable.

**Table 4:** Correlation analysis (Pearson *r* and two-tailed *P* value) among variables in Survey 3 (N=381).

Variable			Identification with social environment		Identification with government		Identification with people around the world (control)		Trust		App acceptance (more app functions)		Installation of app (0=no, 1=yes/no, unsure=missing)
**Identification with social environment**													
	*r*	1		0.08		0.25		0.18		0.09		0.08	
	*P* value	—^a^		.14		<.001		<.001		.07		.14	
**Identification with government**													
	*r*	0.08		1		0.36		0.57		0.26		0.14	
	*P* value	.14		—		<.001		<.001		<.001		.006	
**Identification with people around the world (control)**													
	*r*	0.25		0.36		1		0.22		0.05		–0.03	
	*P* value	<.001		<.001		—		<.001		.34		.54	
**Trust**													
	*r*	0.18		0.57		0.22		1		0.49		0.30	
	*P* value	<.001		<.001		<.001		—		<.001		<.001	
**App acceptance (more app functions)**													
	*r*	0.09		0.26		0.05		0.49		1		0.47	
	*P* value	.07		<.001		.34		<.001		—		<.001	
**Installation of app (0=no, 1=yes/no)**													
	*r*	0.08		0.14		–0.03		0.30		0.47		1	
	*P* value	.14		.006		.54		<.001		<.001		—	

^a^Not applicable.

### Testing Hypotheses

**Figure 2:**
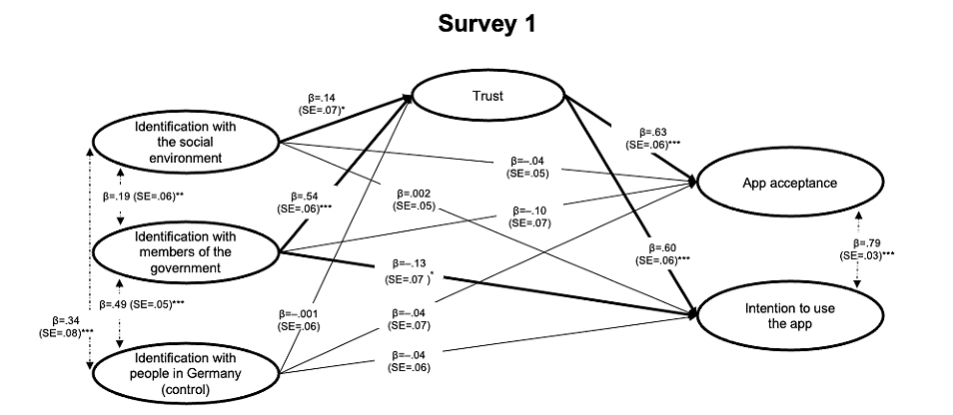
Structural equation model tested in Survey 1 (prelaunch, May 2020; N=355). Indirect effects via trust are reported in Table 6. Coefficients are fully standardized (MPlus STDYX standardization). SE: Standard Error. *P<.05; **P<.01; ***P<.001.

**Figure 3:**
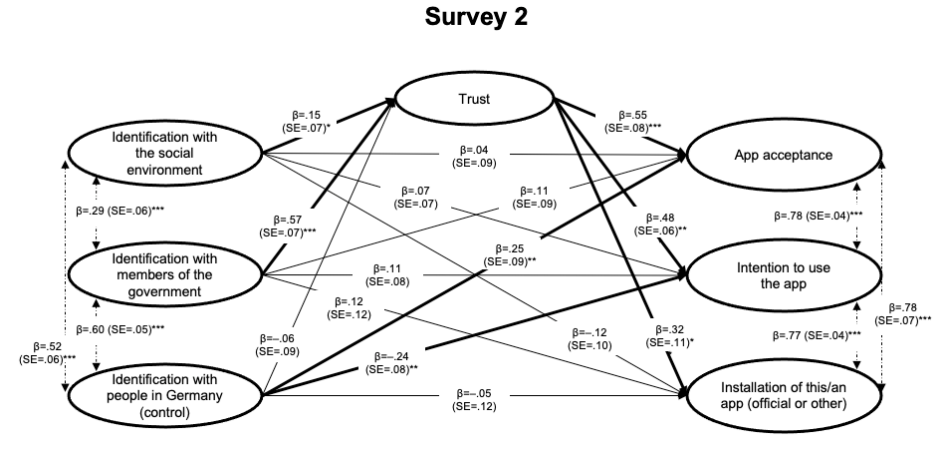
Structural equation model tested in Survey 2 (right after the app was launched, June 2020; N=308). Indirect effects via trust are reported in Table 6. Coefficients are fully standardized (MPlus STDYX standardization). SE: Standard Error. *P<.05; **P<.01; ***P<.001.

**Figure 4:**
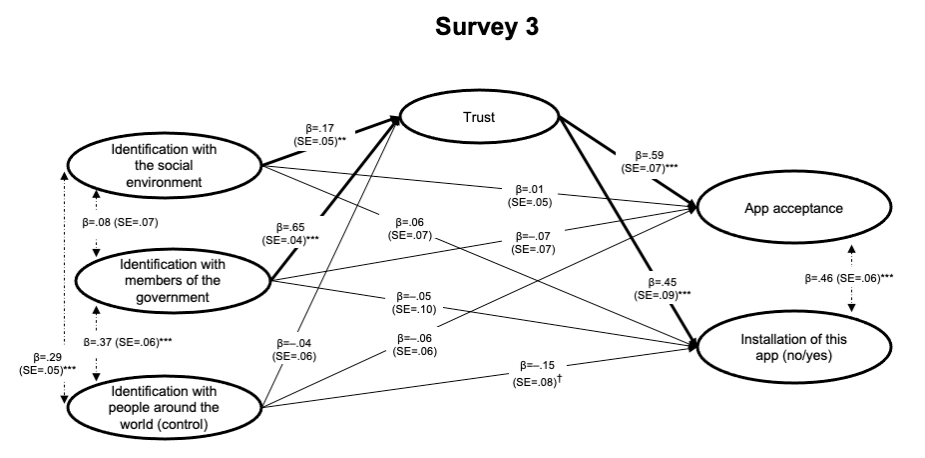
Structural equation model tested in Survey 3 (after launch of the app in December 2020, during discussion about adding more functions and collecting more personal data; N=381). Indirect effects via trust are reported in Table 6. Coefficients are fully standardized (MPlus STDYX standardization). SE: Standard Error. †*P*<.10; **P<.01; ***P<.001.

The results of the structural model for each survey are presented in Figures 2-4. For the interested reader, results from the measurement model (factor loadings for individual items) are presented in Table S1 in Multimedia Appendix 1. Overall, the results indicated a good model fit across studies (Table 5). Note that the models reported here tested the predictions as outlined above and were *not* optimized to improve model fit in any way (eg, based on modification indices); for the interested reader, an alternative model for each survey, which (1) excluded the respective control variable (identification with people in Germany/around the world) and (2) included correlations between specific error terms, did improve model fit across studies above 0.90 (see Table S3 in Multimedia Appendix 1).

**Table 5:** Tests of model fit and fit indices for (nonoptimized) models tested across the three surveys.

Survey	*χ2* (*df*)	*P* value	CFI^a^	TLI^b^	RMSEA^c^	SRMR^d^
1	1339.486 (363)	<.001	0.86	0.85	0.087	0.062
2	716.011 (386)	<.001	0.84	0.82	0.053	0.060
3	719.157 (420)	<.001	0.86	0.85	0.043	0.054

^a^CFI: comparative fit index.

^b^TLI: Tucker Lewis index.

^c^RMSEA: root mean square error of approximation.

^d^SRMR: standardized root mean square residual.

**Table 6:** Indirect effects of identification on app-related outcomes via trust across the three surveys.

Predictor		Outcome	Indirect effect via trust	
			b (SE)^a^	*P* value
**Survey 1**				
	ID^b^ with social environment	App acceptance	0.088 (0.044)	.046
	ID with government	App acceptance	0.343 (0.051)	<.001
	ID with social environment	Intention to use the app	0.083 (0.042)	.047
	ID with government	Intention to use the app	0.325 (0.049)	<.001
**Survey 2**				
	ID with social environment	App acceptance	0.081 (0.040)	.04
	ID with government	App acceptance	0.314 (0.057)	<.001
	ID with social environment	Intention to use the app	0.070 (0.035)	.04
	ID with government	Intention to use the app	0.272 (0.046)	<.001
	ID with social environment	Installation of this/an app	0.048 (0.029)	.10
	ID with government	Installation of this/an app	0.185 (0.067)	.006
**Survey 3**				
	ID with social environment	App acceptance	0.098 (0.032)	.002
	ID with government	App acceptance	0.382 (0.049)	<.001
	ID with social environment	Installation of this app	0.074 (0.025)	.004
	ID with government	Installation of this app	0.288 (0.061)	<.001

^a^Coefficients are fully standardized (MPlus STDYX standardization).

^b^ID: identification.

Moreover, when testing for *indirect effects* of the identification measures (identification with the government and with the social environment) predicting outcomes regarding the app via greater trust, all indirect effects were supported (Table 6). This indicates that both types of identification (not only identification with members of the government but also identification with the social environment) predicted more trust and, accordingly, greater acceptance toward intentions to use or use (installation) of the app. In short, the results supported H1 and H2 across the three surveys and these central points in time, further demonstrating that the relation hypothesized in H2 was mediated via trust.

## Discussion

### Principal Results

In this study, we focused on the question: When are people motivated to use a contact tracing app? Results across three surveys at different points in time demonstrated the role of the social groups people belong to: the more people identified with their social environment (the beneficiaries) and the more they identified with members of the government (the source), the greater their app acceptance (ie, intentions and app installation). As predicted, identification with members of the government predicted greater app acceptance via more trust in the government; this outlines that trust in the source may be an important aspect that contributes to the acceptance of new technology, and that identification with the source may serve as a predictor of said trust. As such, the findings demonstrate the importance of these social groups beyond other target groups of identification (ie, people living in Germany or people around the world more generally).

Interestingly, the relation between identification with the social environment (ie, beneficiaries) and more app acceptance also seemed to be mediated via greater *trust*. To speculate about this exploratory finding, as highlighted in the Introduction, it is possible that identification with the social environment may not only make people more willing to trust those who explicitly belong to their ingroup (ie, social environment) but potentially even those who act as representatives for their larger ingroup (eg, society). However, this assumption awaits further confirmatory testing.

### Limitations

Notably, our work is not without limitations. First, our study followed a cross-sectional design. Accordingly, the data presented here (including the mediation analyses) do not allow for conclusions about causality. An important step for future work is to go beyond this approach via collecting longitudinal data, which can not only help to investigate how the relations between these concepts unfold over time but can also enable examining how intentions and levels of social identification may change (eg, over the course of a pandemic or changes in the contact tracing app). Second, although we assessed data at different points in time and replicated the same model across three surveys, we did so only within a sample from one culture (people living in Germany). Accordingly, replicating this work with experimental manipulations and in different societies would be desirable. Third, this work focused on intentions to use and use of the app, and the latter was operationalized via having installed the app (but not necessarily whether people constantly let it trace their movements); this served to be consistent across studies in our measures (as in Surveys 1 and 2, the required features of the upcoming app, such as keeping their Bluetooth on, were still unclear). Although these are important outcomes to study, it would be useful to build upon this work and extend it to, for instance, whether users do insert a (positive) test result.

### Implications

Many new measures have been implemented since the outbreak of COVID-19 in 2020. The effectiveness of such measures greatly depends on people’s willingness to adhere to them. Prior work suggests that one way to contribute to the acceptance of new measures is to appeal to *personal* benefits and keep the personal costs as low as possible. This study extends this prior work by adopting a focus on social relationships, namely, the extent to which people *identify with* (and trust in) the social groups that are relevant in this regard. The set of findings is relevant both in theoretical and practical terms.

From a theoretical point of view, these results add a crucial aspect to existing models on technology acceptance and health beliefs [15,46]. The results highlight that beyond known personal motives (eg, individual benefits and barriers), it is also important to take *social* aspects (eg, collective benefits or barriers) into account to understand when and why people will adopt a new technology. This seems especially relevant in interdependent contexts (eg, a pandemic) in which one person’s health-related behavior (eg, social distancing, hand hygiene, or contact-tracing app use) more directly affects other people’s situation. In line with prior work, we found that trust in the government constitutes a determinant of app acceptance [10,36] and, importantly, our findings show that identification predicts said trust.

This result is also important from a practical point of view. It suggests that to motivate people to adopt new technology such as the COVID-19 tracing app, one may not need to know all *personal* and facilitatory (which typically vary between people) barriers; rather, it seems important to foster their identification with social groups involved in the process. Indeed, promoting (the salience of) social identification with the source and/or beneficiaries could be achieved via several means. One example is to report more about “collective success” (eg, in fighting the pandemic; see related research on collective pride [47]). A second example is via governmental leaders engaging in identity leadership (creating a shared sense of “us” [48-50]) or politicians using consensual communication and/or “we”-referencing language (ie, referring more to “we,” “us,” and “ours,” suggesting that they see themselves and act as “our leaders” [51,52]).

### Conclusion

The results suggest that to motivate people to adhere to new measures in times of crisis (a global pandemic in this case), it is important to, in a responsible manner, take their relationships to social groups (ie, their *identification* with the source as well as the beneficiaries) into account (eg, potentially appealing to people’s identification with these social groups). Doing so may not only contribute to a better understanding of what motivates people to accept new measures but also what contributes to their actual willingness to follow through with them.

## Appendix

Multimedia Appendix 1 Items, factor loadings, alternative models tested, and measures across all studies in their original order (Tables S1-S3).

Multimedia Appendix 2 Original materials (in German) for Surveys 1-3.
