# Supplementary material for: How Identification With the Social Environment and With the Government Guide the Use of the Official COVID-19 Contact Tracing App: Three Quantitative Survey Studies
Source: JMIR Mhealth Uhealth. 2021 Nov 24;9(11):e28146. doi: 10.2196/28146 (PMC8614392; doi:10.2196/28146)
Supplement: Multimedia Appendix 1 [file mhealth_v9i11e28146_app1.pdf]

Supplement: Item loadings, alternative models, overview of measures

How identification with the social environment and with the government guide the use of the official COVID-19 contact tracing app: Three quantitative survey studies

Annika Scholl & Kai Sassenberg

Table S1. Items on identification across studies and factor loadings for each item on the respective identification factor in the measurement model

Identification with the personal environment

Item wording	Item	Study 1 Estimate	Study 1 *SE*	Study 2 Estimate	Study 2 *SE*	Study 3 Estimate	Study 3 *SE*
How much do you have in common with the following groups? People in my personal environment	IDU1	0.678	0.036	0.717	0.052	0.751	0.049
How strongly do you feel that you belong to the following groups? People in my personal environment	IDU2	0.815	0.046	0.874	0.032	0.740	0.043
How strongly do you feel connected to the following groups? People in my personal environment	IDU3	0.876	0.026	0.840	0.046	0.823	0.041
How much solidarity to you experience with the following groups? People in my personal environment	IDU4	0.801	0.041	0.789	0.051	0.779	0.039
How much do you identify with the following groups (i.e., experience belongingness, affection, care about them)? People in my personal environment	IDU5	0.829	0.033	0.877	0.035	0.796	0.046
How concerned do you feel if something bad would happen to each of the following groups? People in my personal environment	IDU6	0.627	0.081	0.619	0.060	0.517	0.073

Identification with people in Germany (Studies 1+2) vs. Humanity (Study 3)

Item wording	Item	Study 1 Estimate	Study 1 *SE*	Study 2 Estimate	Study 2 *SE*	Study 3 Estimate	Study 3 *SE*
How much do you have in common with the following groups? People living in Germany // People around the world	IDD1	0.573	0.061	0.669	0.050	0.680	0.049
How strongly do you feel that you belong to the following groups? People living in Germany // People around the world	IDD2	0.802	0.027	0.776	0.043	0.831	0.034
How strongly do you feel connected to the following groups? People living in Germany // People around the world	IDD3	0.835	0.030	0.798	0.034	0.840	0.032
How much solidarity to you experience with the following groups? People living in Germany // People around the world	IDD4	0.705	0.038	0.812	0.039	0.855	0.040
How much do you identify with the following groups (i.e., experience belongingness, affection, care about them)? People living in Germany // People around the world	IDD5	0.773	0.031	0.762	0.041	0.803	0.033
How concerned do you feel if something bad would happen to each of the following groups? People living in Germany // People around the world	IDD6	0.631	0.039	0.526	0.064	0.658	0.043

Identification with members from the government

Item wording	Item	Study 1 Estimate	Study 1 *SE*	Study 2 Estimate	Study 2 *SE*	Study 3 Estimate	Study 3 *SE*
How much do you have in common with the following groups? Members of the government	IDR1	0.715	0.033	0.795	0.040	0.743	0.038
How strongly do you feel that you belong to the following groups? Members of the government	IDR2	0.744	0.036	0.655	0.051	0.595	0.051
How strongly do you feel connected to the following groups? Members of the government	IDR3	0.834	0.024	0.781	0.036	0.804	0.033
How much solidarity to you experience with the following groups? Members of the government	IDR4	0.750	0.029	0.923	0.029	0.883	0.031
How much do you identify with the following groups (i.e., experience belongingness, affection, care about them)? Members of the government	IDR5	0.762	0.031	0.713	0.043	0.726	0.039
How concerned do you feel if something bad would happen to each of the following groups? Members of the government	IDR6	0.604	0.044	0.613	0.057	0.674	0.051

Note. Coefficients are fully standardized (MPlus STDYX standardization)

Table S2. Items on trust and outcome across studies and factor loadings for each item on the respective factor (trust or outcome) in the measurement model

Trust in the government

Item wording	Item	Study 1 Estimate	Study 1 *SE*	Study 2 Estimate	Study 2 *SE*	Study 3 Estimate	Study 3 *SE*
How trustworthy do you evaluate the government to be?	TR1	0.902	0.018	0.949	0.024	0.920	0.022
How honest do you evaluate the government to be?	TR2	0.896	0.013	0.835	0.032	0.836	0.029
How competent do you evaluate the government to be?	TR3	0.733	0.032	0.792	0.035	0.725	0.043
How reliable do you evaluate the government to be?	TR4	0.905	0.017	0.941	0.023	0.865	0.024

App acceptance - before and right after the launch of the official Corona-App (low privacy infringement)

Item wording	Item	Study 1 Estimate	Study 1 *SE*	Study 2 Estimate	Study 2 *SE*	Study 3 Estimate	Study 3 *SE*
I find it acceptable that such an app should be used.	ACC1	0.872	0.020	0.888	0.032		
Using such an app would invade on my privacy.	ACC2	0.796	0.024	0.648	0.051		
I have little reason to be concerned about my privacy due to this app.	ACC3	0.712	0.042	0.709	0.064	n.a.	
The fact that this app could be used makes me uneasy.	ACC4	0.814	0.025	0.701	0.046		
I can completely understand why this app should be in use.	ACC5	0.689	0.037	0.737	0.051		
Because of this app, I feel like someone is always watching me.	ACC6	0.815	0.025	0.758	0.040		

App acceptance - with regard to new app functions collecting more data (low privacy infringement)

Item wording	Item	Study 1 Estimate	Study 1 *SE*	Study 2 Estimate	Study 2 *SE*	Study 3 Estimate	Study 3 *SE*
I would be ready to reveal more of myself to provide a basis that enables future decisions.	ACN1					0.966	0.023
I would feel uncomfortable to provide more information about myself, (even) if it concerns decisions about societal life.	ACN2					0.837	0.032
I would not be concerned about providing more data about myself to enable future decision-making.	ACN3	n.a.		n.a.		0.843	0.033
I would be okay to give more personal data to provide a basis for the measures.	ACN4					0.947	0.025
I would feel like someone is watching me if I provided more data, irrespective of how this data would be used.	ACN5					0.709	0.043
I would feel uneasy when giving up more personal data that will be used for decision-making.	ACN6					0.735	0.049
It would invade my privacy to provide my data (also) to include them in these measures.	ACN7					0.640	0.050
I would be fine if more data from myself would be stored for such a data basis.	ACN8					0.867	0.030

Note. Coefficients are fully standardized (MPlus STDYX standardization)

Table S3. Alternative models to improve model fit (based on modification indices)

Study	χ^2	df	*P*-value	CFI	TLI	RMSEA	SRMR
1	546.185	218	<.001	.94	.93	.065	.054
2	347.915	235	<.001	.93	.82	.039	.044
3	374.082	263	<.001	.92	.93	.033	.045

Model adaptations:
Study 1: three errors correlating (IDU6 with IDU4; ACC5 with ACC1; IDR3 with IDR2) and excluding control variable identification with people living in Germany;
Study 2: four 4 error terms correlating (ACC5 with ID with personal environment; ACC5 with ID with government; IDU6 with app acceptance; IDR2 with ID with government) and excluding control variable identification with people living in Germany;
Study 3: three error terms correlating (IDU1 with ID with personal environment; TR3 with Trust; IDU6 with IDU5); and excluding control variable identification with people around the world

Measures assessed in all studies in their original order:

- Study 1:
social identification; app acceptance (low privacy infringement); appraisal of the app purpose (here, exploratory); trust in the government; exploratory other measures for another study (trust in other organizations; appraisal of the government measures; motives to use a Corona-App); willingness to use the Corona-App; having already installed a different app related to Corona; perceived norms about hand-washing, the Corona-App, and physical distancing; acceptance of further measures in terms of surveillance via drones; conspiracy theories (general and related to Corona); perceived threat due to Corona; extent to which their personal lives had changed
- Study 2:
manipulated identity salience (3 between-conditions: (1) identification with the government; (2) identification with the social environment; (3) control condition with self-affirmation = personal identity salient); participants here were asked to recount a situation in which they had been satisfied with either the government, their social environment, or a personal competency they had; app acceptance (low privacy infringement); appraisal of the app purpose (here, exploratory); trust in the government; willingness to use the Corona-App; having already installed a different app related to Corona; acceptance of further measures in terms of surveillance via drones (exploratory); a manipulation check; social identification with their social environment, people living in Germany, members of the government, and (exploratory) scientists from the health domain; perceived threat due to Corona; extent to which their personal lives had changed
- Study 3:
manipulated identity salience (2 between-conditions: (1) identification with the government; (2) control condition = personal identity salient); participants here were asked to recount a situation in which they had been satisfied with either the government or with their personal way of handling things regarding the pandemic; manipulation check; app acceptance (low privacy infringement) with regard to new functions; perceived identity entrepreneurship by the government (here, exploratory); trust in the government; whether or not they had already installed and were using the Corona-App; social identification with members of the government, their social environment, people around the world (humanity); perceived threat due to Corona; extent to which their personal lives had changed
