# Supplementary material for: How Identification With the Social Environment and With the Government Guide the Use of the Official COVID-19 Contact Tracing App: Three Quantitative Survey Studies
Source: JMIR Mhealth Uhealth. 2021 Nov 24;9(11):e28146. doi: 10.2196/28146 (PMC8614392; doi:10.2196/28146)
Supplement: Multimedia Appendix 2 [file mhealth_v9i11e28146_app2.pdf]

Supplement: Materials

How identification with the social environment and with the government guide the use of the official COVID-19 contact tracing app: Three quantitative survey studies

Annika Scholl & Kai Sassenberg

Study 1 Measures

Social Identification

1. Wie viel haben Sie Ihrer Meinung nach mit den folgenden Gruppen gemeinsam?
2. Wie stark fühlen Sie sich den folgenden Gruppen zugehörig?
3. Wie sehr fühlen Sie sich mit den folgenden Gruppen verbunden?
4. Wie viel Solidarität empfinden Sie mit den folgenden Gruppen?
5. Wie sehr identifizieren Sie sich mit der jeweiligen Gruppe (d.h. fühlen sich zugehörig zu ihr, empfinden Zuneigung, sorgen sich um diese)?
6. Wie betroffen fühlen Sie sich, wenn einer der folgenden Gruppen etwas Schlimmes passiert?

Scale:

	1 - gar nichts /keine	2	3	4	5	6	7 – sehr viel/ sehr stark
Menschen in meinem persönlichen Umfeld	○	○	○	○	○	○	○
In Deutschland lebende Menschen	○	○	○	○	○	○	○
Mitglieder der Regierung	○	○	○	○	○	○	○
WissenschaftlerInnen im Gesundheitsbereich	○	○	○	○	○	○	○

App Acceptance (low privacy infringement)

Zur Eindämmung der Verbreitung von Covid-19 werden aktuell Maßnahmen diskutiert, die die Zurückverfolgung oder den Austausch von Bewegungen der BürgerInnen (tracing) beinhalten könnten.

Aktuell plant die Regierung die Entwicklung einer Corona-App, die (z.B. über Handy-Ortung oder Bluetooth) Corona-Ansteckungen nachverfolgen und HandybesitzerInnen "warnen" könnte, wenn sich eine infizierte Person in der Nähe befindet oder der/die HandybesitzerIn mit einer infizierten Person Kontakt hatte. Die Verwendung der App soll freiwillig sein.

Wie bewerten Sie persönlich den Aufruf zur Nutzung dieser geplanten Tracing-App?

1. Ich finde es akzeptabel, dass eine solche App genutzt werden soll.
2. Die Nutzung einer solchen App schränkt meine Privatsphäre ein.
3. Ich habe wenig Grund, mir wegen dieser App Sorgen um meine Privatsphäre zu machen.
4. Die Tatsache, dass diese App genutzt werden könnte, verunsichert mich.
5. Ich kann vollkommen nachvollziehen, warum es diese App geben soll.
6. Durch diese App fühle ich mich, als könnte mich ständig jemand beobachten.

Scale:

- ◯ 1 - trifft gar nicht zu (1)
- ◯ 2 (2)
- ◯ 3 (3)
- ◯ 4 (4)
- ◯ 5 (5)
- ◯ 6 (6)
- ◯ 7 - trifft voll zu (7)

Exploratory: appraisal of the app

Wozu denken Sie, werden diese Tracing-App und Ihre Daten tatsächlich verwendet?

	1 - trifft gar nicht zu	2	3	4	5	6	7 - trifft voll zu
Um die Verbreitung des Virus zu stoppen	○	○	○	○	○	○	○
Zum Wohle der Sicherheit anderer Personen	○	○	○	○	○	○	○
Um die Verbreitung des Virus besser zu verstehen	○	○	○	○	○	○	○
Um Fehlverhalten von Menschen zu verhindern	○	○	○	○	○	○	○
Zum Wohle meiner eigenen Sicherheit	○	○	○	○	○	○	○
Um Menschen zu überwachen	○	○	○	○	○	○	○

Trust in the government

Bitte geben Sie an, wie Sie die Regierung in Bezug auf das Einsetzen dieser App und deren Daten einschätzen würden.

1. Wie vertrauenswürdig schätzen Sie die Regierung ein?
2. Wie ehrlich schätzen Sie die Regierung ein?
3. Wie kompetent schätzen Sie die Regierung ein?
4. Wie glaubwürdig schätzen Sie die Regierung ein?

Scale:

○ 1 - gar nicht

○ 2

○ 3

○ 4

○ 5

○ 6

○ 7 - sehr

Exploratory other measures

(a) Trust in other organizations

Wie sehr vertrauen Sie den unten genannten Personen und Organisationen?

	1 - sehr geringes Vertrauen	2	3	4	5	6	7 - sehr starkes Vertrauen
Robert-Koch-Institut (Bundesbehörde für Krankheitsüberwachung und -prävention)	○	○	○	○	○	○	○
Bundeszentrale für gesundheitliche Aufklärung	○	○	○	○	○	○	○
Länderministerien	○	○	○	○	○	○	○
Bundesministerien	○	○	○	○	○	○	○

(b) appraisal of the government measures

Wie nehmen Sie aktuell das Vorgehen der Regierung in der Bekämpfung der Pandemie wahr?

	1 - gar nicht	2	3	4	5	6	7 - sehr
An den Interessen der BürgerInnen orientiert	○	○	○	○	○	○	○
Umsichtig	○	○	○	○	○	○	○
Repräsentativ dafür, was vielen BürgerInnen wichtig ist	○	○	○	○	○	○	○
An Sicherheit orientiert	○	○	○	○	○	○	○
Zentrale ethische Standards einhaltend	○	○	○	○	○	○	○
Transparent	○	○	○	○	○	○	○
An Fairness orientiert	○	○	○	○	○	○	○
Verantwortungsvoll	○	○	○	○	○	○	○

(c) motives to use a Corona-App

Ich bin/wäre bereit, diese Tracing-App zu nutzen…

	1 - trifft gar nicht zu	2	3	4	5	6	7 - trifft voll zu
… um mich selbst vor einer Infektion mit Covid-19 zu schützen.	○	○	○	○	○	○	○
... um für meinen Beitrag zur Forschung Anerkennung zu erhalten.	○	○	○	○	○	○	○
… um besonders gefährdete Gruppen vor einer Ansteckung zu schützen.	○	○	○	○	○	○	○
… um nicht der/die einzige zu sein, der/die diese Apps nicht nutzt.	○	○	○	○	○	○	○
… um das Gesundheitssystem vor einer Überlastung zu schützen.	○	○	○	○	○	○	○
… um meine nahen Angehörigen (Eltern, Großeltern) vor einer Infektion zu schützen.	○	○	○	○	○	○	○

Willingness to use the Corona-App

In welchem Ausmaß sind/wären Sie bereit, diese Tracing-App zu nutzen?

- ◯ 1 - gar nicht
- ◯ 2
- ◯ 3
- ◯ 4
- ◯ 5
- ◯ 6
- ◯ 7 - voll und ganz

Having already installed a different app related to Corona

Haben Sie bereits eine andere Corona App auf Ihrem Handy installiert?

- ◯ ja
- ◯ nein

Perceived norms about hand-washing, the app, and physical distancing

Was denken Sie: Wie viele Personen in Ihrem Umfeld würden den folgenden Aussagen zustimmen?

	niemand (0%) 0	25	50	75	alle (100%) 100
Man sollte sich häufig und gründlich die Hände waschen.					
Man sollte Corona-tracing Apps nutzen, um zur Prävention beizutragen.					
Man sollte zuhause bleiben, um direkte Kontakte mit anderen zu minimieren.					

Acceptance of further measures in terms of surveillance via drones

In welchem Ausmaß sind/wären Sie bereit, zur Bekämpfung der Ausbreitung des Virus auch weitere digitale Maßnahmen zum Tracing, wie beispielsweise den Einsatz von Drohnen, zu akzeptieren?

- ○ 1 - gar nicht
- ○ 2
- ○ 3
- ○ 4
- ○ 5
- ○ 6
- ○ 7 - voll und ganz

Exploratory: conspiracy theories

Wie sehr stimmen Sie der folgenden Aussage zu?
Ich wäre zurückhaltend, irgendwelche groß angelegten Veränderungen der gesellschaftlichen Ordnung durchzuführen.

- ◯ 1 - stimme gar nicht zu
- ◯ 2
- ◯ 3
- ◯ 4
- ◯ 5
- ◯ 6
- ◯ 7 - stimme voll zu

Bitte geben Sie an, inwiefern Sie den folgenden Aussagen über das Coronavirus zustimmen.

1. Die Nachrichten übertreiben die Zahlen und die Gefahr von Covid-19.
2. Mächtige Leute benutzen Covid-19, um der Wirtschaft zu schaden.
3. Die Panik über Covid-19 wird teilweise durch Menschen verursacht, die dem politischen System schaden wollen.
4. Es ist wichtiger, an die Wirtschaft zu denken statt Panik zu machen wegen eines Virus, das letztlich nicht so gefährlich ist.
5. Covid-19 ist nur eine Möglichkeit der Regierung, die Macht der kleinen Leute einzuschränken.
6. Es geschehen sehr viele wichtige Dinge in der Welt, über die die Öffentlichkeit nie informiert wird.
7. Die da oben machen ja eh was sie wollen.
8. Ein paar mächtige Personengruppen bestimmen über das Schicksal von Millionen von Menschen.
9. Es gibt geheime Organisationen, die großen Einfluss auf politische Entscheidungen haben.
10. Die verschiedenen in den Medien zirkulierenden Verschwörungstheorien halte ich für ausgemachten Blödsinn.
11. Politiker und andere Führungspersönlichkeiten sind nur Marionetten der dahinterstehenden Mächte.

12. Die meisten Menschen erkennen nicht, in welchem Ausmaß unser Leben durch Verschwörungen bestimmt wird, die im Geheimen ausgeheckt werden.
13. Es gibt keinen vernünftigen Grund, Regierungen, Geheimdiensten oder Medien zu misstrauen.
14. Die internationalen Geheimdienste mischen viel mehr in alltäglichen Dingen mit, als man denkt.
15. Geheime Organisationen können Leute psychisch so manipulieren, dass diese nicht wissen, dass ihr Leben von außen bestimmt wird.
16. Es gibt bestimmte politische Zirkel, die geheime Pläne verfolgen und sehr viel Einfluss haben.
17. Die meisten Menschen machen sich keine Vorstellung davon, wie sehr unser Leben bestimmt wird von im Geheimen geschmiedeten Plänen.

○ 1 - stimme gar nicht zu

○ 2

○ 3

○ 4

○ 5

○ 6

○ 7 - stimme voll zu

Bei den folgenden Fragen geht es uns um Ihre spontane Einschätzung, nicht um die Meinung von anderen oder der Mehrheit. Bitte geben Sie jeweils an, wie sehr die Aussagen Ihrer Meinung nach zutreffen.

Exploratory: threat due to Corona / extent to which their personal lives have changed

Bitte beantworten Sie abschließend einige Fragen zu den Auswirkungen von Corona auf Ihren Alltag.

Wie besorgt fühlen Sie sich aktuell aufgrund der Corona-Situation?
Wie bedroht fühlen Sie sich aktuell aufgrund der Corona-Situation?
Wie sehr hat sich Ihr aktueller persönlicher Alltag (Freizeit) durch Corona verändert?
Wie sehr hat sich Ihr aktueller Studienalltag durch Corona verändert?

○ 1 - gar nicht

○ 2

○ 3

○ 4

○ 5

○ 6

○ 7 - sehr

Was denken Sie, wird in der Studie untersucht? (optional)

____

____

____

____

____

Demographics:

- Student / employed
- age
- gender
- tongue
- years having lived in Germany

Debriefing and thank you

Study 2 Measures

Manipulated identity salience

Für eine Studie sammeln wir Beispiele für Situationen, die Menschen erlebt haben.
Im Leben gehören wir unterschiedlichen Gruppen an und leben in unterschiedlichen Kontexten, die z.B. durch unser direktes persönliches Umfeld (NachbarInnen, MitbürgerInnen, FreundInnen), durch den Beruf und durch die Gesellschaft und Regierung mit geprägt sind.

3 between conditions:
(1) identification with the government

Für eine Studie sammeln wir Beispiele für Situationen, die Menschen erlebt haben.
Im Leben gehören wir unterschiedlichen Gruppen an und leben in unterschiedlichen Kontexten, die z.B. durch unser direktes persönliches Umfeld (NachbarInnen, MitbürgerInnen, FreundInnen), durch den Beruf und durch die Gesellschaft und Regierung mit geprägt sind.
Im Folgenden wollen wir mehr darüber herausfinden, wie Menschen ihr Leben unter der aktuellen Regierung erleben. Dabei gibt es sicher Situationen, in denen sie sich z.B. ein anderes Vorgehen wünschen; genauso gibt es bestimmt Situationen, in denen Sie froh oder erleichtert darüber sind, unter der aktuellen Regierung zu leben.
An dieser Stelle möchten wir Sie bitten, sich eine persönliche Erfahrung aus Ihrem Leben unter der aktuellen Regierung in Erinnerung zu rufen: Denken Sie bitte an eine konkrete Situation im letzten Jahr, in der Sie froh darüber waren, unter der aktuellen Regierung zu leben. Bitte nehmen Sie sich einen Augenblick Zeit, sich an eine solche Erfahrung zu erinnern. Beschreiben Sie dann bitte kurz: Wie haben Sie sich in der Situation gefühlt? Was ist in der Situation passiert?

(2) identification with the social environment

Für eine Studie sammeln wir Beispiele für Situationen, die Menschen erlebt haben.
Im Leben gehören wir unterschiedlichen Gruppen an und leben in unterschiedlichen Kontexten, die z.B. durch die Gesellschaft und Regierung, durch den Beruf und durch das direkte persönliche Umfeld (z.B. MitbürgerInnen, FreundInnen, NachbarInnen) von Menschen mit geprägt sind.
Im Folgenden wollen wir mehr darüber herausfinden, wie Menschen ihr Leben mit Menschen in ihrem persönlichen Umfeld erleben. Dabei gibt es sicher Situationen, in denen sie sich z.B. ein anderes persönliches Umfeld wünschen; genauso gibt es bestimmt Situationen, in denen Sie froh oder erleichtert darüber sind, in Ihrem aktuellen persönlichen Umfeld zu leben.
An dieser Stelle möchten wir Sie bitten, sich eine persönliche Erfahrung aus Ihrem Leben in Ihrem persönlichen Umfeld in Erinnerung zu rufen: Denken Sie bitte an eine konkrete Situation im letzten Jahr, in der Sie froh darüber waren, in Ihrem aktuellen persönlichen Umfeld zu leben. Bitte nehmen Sie sich einen Augenblick Zeit,

sich an eine solche Erfahrung zu erinnern. Beschreiben Sie dann bitte kurz: Wie haben Sie sich in der Situation gefühlt? Was ist in der Situation passiert?

(3) control condition with self-affirmation

Für eine Studie sammeln wir Beispiele für Situationen, die Menschen erlebt haben. Im Leben haben wir bereits manche Kompetenzen inne und lernen wiederum andere, neue Fähigkeiten hinzu.
Im Folgenden wollen wir mehr darüber herausfinden, wie Menschen ihre eigenen Kompetenzen erleben. Dabei gibt es sicher Kompetenzen, die Sie sich noch mehr aneignen möchten; genauso gibt es bestimmt Stärken, bei denen Sie froh oder erleichtert darüber sind, diese bereits im aktuellen Ausmaß zu besitzen.
An dieser Stelle möchten wir Sie bitten, sich eine persönliche Stärke in Erinnerung zu rufen: Denken Sie bitte an eine konkrete Situation im letzten Jahr, in der Sie froh darüber waren, diese persönliche Stärke zu besitzen. Bitte nehmen Sie sich einen Augenblick Zeit, sich an eine solche Erfahrung zu erinnern. Beschreiben Sie dann bitte kurz: Wie haben Sie diese Stärke erlebt? Was genau bedeutet diese Stärke für Sie?

app acceptance (low privacy infringement)

Im Folgenden interessieren wir uns für Themen rund um Corona. Bitte geben Sie ganz spontan an, wie sehr die jeweiligen Aussagen auf Sie zutreffen - es gibt kein richtig und falsch.

Zur Eindämmung der Verbreitung von Covid-19 wurden aktuell neue Maßnahmen implementiert, die die Zurückverfolgung oder den Austausch von Bewegungen der BürgerInnen (tracing) beinhalten. Dazu hat die Regierung die Entwicklung einer Corona-App in Auftrag gegeben, die (z.B. über Bluetooth) Corona-Ansteckungen nachverfolgen und HandybesitzerInnen "warnen" kann, wenn sich eine infizierte Person in der Nähe befindet oder der/die HandybesitzerIn mit einer infizierten Person Kontakt hatte. Die App steht seit 16.06. zur Verfügung und ihre Verwendung ist freiwillig.

Wie bewerten Sie persönlich den Aufruf zur Nutzung dieser Corona-App?

1. Ich finde es akzeptabel, dass eine solche App genutzt werden soll
2. Die Nutzung einer solchen App schränkt meine Privatsphäre ein
3. Ich habe wenig Grund, mir wegen dieser App Sorgen um meine Privatsphäre zu machen
4. Die Tatsache, dass diese App genutzt werden könnte, verunsichert mich
5. Ich kann vollkommen nachvollziehen, warum es diese App geben soll
6. Durch diese App fühle ich mich, als könnte mich ständig jemand beobachten.

- ○ 1 - trifft gar nicht zu
- ○ 2
- ○ 3
- ○ 4
- ○ 5
- ○ 6
- ○ 7 - trifft voll zu

appraisal of app purpose (exploratory)

Wozu denken Sie, werden diese Corona-App und Ihre Daten tatsächlich verwendet?

	1 - trifft gar nicht zu	2	3	4	5	6	7 - trifft voll zu
Um die Verbreitung des Virus zu stoppen	○	○	○	○	○	○	○
Zum Wohle der Sicherheit anderer Personen	○	○	○	○	○	○	○
Um die Verbreitung des Virus besser zu verstehen	○	○	○	○	○	○	○
Um Fehlverhalten von Menschen zu verhindern	○	○	○	○	○	○	○
Zum Wohle meiner eigenen Sicherheit	○	○	○	○	○	○	○
Um Menschen zu überwachen	○	○	○	○	○	○	○

Trust in the government

Bitte geben Sie an, wie Sie die Regierung in Bezug auf das Einsetzen dieser App und deren Daten einschätzen würden.

5. Wie vertrauenswürdig schätzen Sie die Regierung ein?
6. Wie ehrlich schätzen Sie die Regierung ein?
7. Wie kompetent schätzen Sie die Regierung ein?
8. Wie glaubwürdig schätzen Sie die Regierung ein?

Scale:

- ◯ 1 - gar nicht
- ◯ 2
- ◯ 3
- ◯ 4
- ◯ 5
- ◯ 6
- ◯ 7 - sehr

willingness to install the corona-app

In welchem Ausmaß sind Sie bereit, diese Tracing-App zu nutzen?

- ◯ 1 - gar nicht
- ◯ 2
- ◯ 3
- ◯ 4
- ◯ 5
- ◯ 6
- ◯ 7 - voll und ganz

having already installed a different app related to corona

Haben Sie bereits diese oder eine andere Corona App auf Ihrem Handy installiert?

- ◯ ja
- ◯ nein

Acceptance of further measures in terms of surveillance via drones

In welchem Ausmaß sind/wären Sie bereit, zur Bekämpfung der Ausbreitung des Virus auch weitere digitale Maßnahmen zum Tracing, wie beispielsweise den Einsatz von Drohnen, zu akzeptieren?

- ○ 1 - gar nicht
- ○ 2
- ○ 3
- ○ 4
- ○ 5
- ○ 6
- ○ 7 - voll und ganz

Manipulation check

Wenn Sie an den ersten Teil des Fragebogens denken, in dem Sie eine Situation notiert haben, in der Sie froh darüber waren, unter der aktuellen Regierung / in Ihrem aktuellen persönlichen Umfeld zu leben - wie sehr fühlen Sie sich jetzt gerade mit den folgenden Gruppen verbunden?

	1 - gar nicht	2	3	4	5	6	7 - sehr stark
Menschen in meinem persönlichen Umfeld	○	○	○	○	○	○	○
Mitglieder der Regierung	○	○	○	○	○	○	○

Wenn Sie an den ersten Teil des Fragebogens denken, in dem Sie sich eine konkrete Situation in Erinnerung gerufen haben, in der Sie froh darüber waren, diese persönliche

Stärke zu besitzen - wie sehr fühlen Sie sich jetzt gerade mit den folgenden Gruppen verbunden?

	1 - gar nicht	2	3	4	5	6	7 - sehr stark
Menschen in meinem persönlichen Umfeld	○	○	○	○	○	○	○
Mitglieder der Regierung	○	○	○	○	○	○	○

Social Identification

7. Wie viel haben Sie Ihrer Meinung nach mit den folgenden Gruppen gemeinsam?
8. Wie stark fühlen Sie sich den folgenden Gruppen zugehörig?
9. Wie sehr fühlen Sie sich mit den folgenden Gruppen verbunden?
10. Wie viel Solidarität empfinden Sie mit den folgenden Gruppen?
11. Wie sehr identifizieren Sie sich mit der jeweiligen Gruppe (d.h. fühlen sich zugehörig zu ihr, empfinden Zuneigung, sorgen sich um diese)?
12. Wie betroffen fühlen Sie sich, wenn einer der folgenden Gruppen etwas Schlimmes passiert?

Scale:

	1 - gar nichts /keine	2	3	4	5	6	7 – sehr viel/ sehr stark
Menschen in meinem persönlichen Umfeld	○	○	○	○	○	○	○
In Deutschland lebende Menschen	○	○	○	○	○	○	○
Mitglieder der Regierung	○	○	○	○	○	○	○
WissenschaftlerInnen im Gesundheitsbereich	○	○	○	○	○	○	○

Exploratory: threat due to Corona / extent to which their personal lives have changed

Bitte beantworten Sie abschließend einige Fragen zu den Auswirkungen von Corona auf Ihren Alltag.

Wie besorgt fühlen Sie sich aktuell aufgrund der Corona-Situation?
Wie bedroht fühlen Sie sich aktuell aufgrund der Corona-Situation?
Wie sehr hat sich Ihr aktueller persönlicher Alltag (Freizeit) durch Corona verändert?
Wie sehr hat sich Ihr aktueller Studienalltag durch Corona verändert?

- ○ 1 - gar nicht
- ○ 2
- ○ 3
- ○ 4
- ○ 5
- ○ 6
- ○ 7 - sehr

Was denken Sie, wird in der Studie untersucht? (optional)

____

____

____

____

____

Demographics:

- Student / employed
- age
- gender
- tongue
- years having lived in Germany

Debriefing and thank you

Study 3 Measures

Manipulated identity salience

(1) identification with the government

Für eine Studie sammeln wir Beispiele für Situationen, die Menschen erlebt haben.

Im Leben befinden wir uns in unterschiedlichen Kontexten, die z.B. durch unseren eigenen Umgang mit Situationen geprägt sind, aber auch durch die Gesellschaft und Regierung. Im Folgenden wollen wir mehr darüber herausfinden, wie Menschen aktuelle Maßnahmen der **Regierung** bezüglich Covid-19 erleben. Dabei gibt es sicher Situationen, in denen Sie sich z.B. ein anderes Vorgehen wünschen; genauso gibt es bestimmt Situationen, in denen Sie erleichtert über manche Entscheidungen sind.

Im Folgenden bitten wir Sie: Denken Sie bitte an eine konkrete Situation, in der Sie **zufrieden mit den Maßnahmen der Regierung** waren. Bitte nehmen Sie sich einen Augenblick Zeit, sich an eine solche Situation zu erinnern. Beschreiben Sie dann bitte kurz: Was ist in der Situation passiert? Wie haben Sie sich in der Situation gefühlt?

(2) identification with self (personal identity salient)

Für eine Studie sammeln wir Beispiele für Situationen, die Menschen erlebt haben.

Im Leben befinden wir uns in unterschiedlichen Kontexten, die z.B. durch die Gesellschaft und Regierung, aber auch durch unseren eigenen Umgang mit Situationen geprägt sind. Im Folgenden wollen wir mehr darüber herausfinden, wie Menschen ihren **eigenen Umgang** mit der aktuellen Situation bezüglich Covid-19 erleben. Dabei gibt es sicher Situationen, in denen Sie sich von sich selbst z.B. ein anderes Vorgehen wünschen; genauso gibt es bestimmt Situationen, in denen Sie erleichtert über manche Entscheidungen oder Handlungen sind, die Sie selbst gezeigt haben.

Im Folgenden bitten wir Sie: Denken Sie bitte an eine konkrete Situation, in der Sie **zufrieden mit ihrem eigenen Umgang mit der aktuellen Situation** waren. Bitte nehmen Sie sich einen Augenblick Zeit, sich an eine solche Situation zu erinnern. Beschreiben Sie dann bitte kurz: Was ist in der Situation passiert? Wie haben Sie sich in der Situation gefühlt?

manipulation check

Wie zufrieden waren Sie in der Situation

- mit sich selbst?
- mit der Regierung?
- mit den äußeren Umständen allgemein?

1 - gar nicht zu zufrieden	2	3	4	5	6	7 - sehr zufrieden

app acceptance (low privacy infringement)

Zur Eindämmung der Verbreitung von Covid-19 hatte die Regierung die Entwicklung einer **Corona-App** beauftragt, die über Bluetooth mögliche Corona-Ansteckungen nachverfolgt und nach dem Kontakt mit möglichen infizierten Personen warnt. Die App steht seit 16.06. zur Verfügung, die BürgerInnen sind durch die Regierung zur freiwilligen Nutzung aufgerufen.

Diese App soll nun um weitere Funktionen ergänzt werden, um womöglich mehr Daten der einzelnen BürgerInnen zu sammeln – z.B. zur genaueren Kontaktnachverfolgung und zur spezifischeren Identifikation von Infektionszeitpunkt und -quelle. Dies soll den Behörden eine größere Datenbasis zur Verfügung stellen, um darauf aufbauend weitere Entscheidungen über Maßnahmen für alle Seiten zu treffen (z.B. Öffung von Restaurants) und Empfehlungen zum individuellen Handeln zu geben.

Im Folgenden interessiert uns: **Wie bewerten Sie spontan diese Überlegungen zur Sammlung zusätzlicher Daten der BürgerInnen über die App?**

	1 - trifft gar nicht zu	2	3	4	5	6	7 - trifft voll zu
Ich wäre bereit, mehr von mir preiszugeben, damit weitere Entscheidungen auf dieser Basis getroffen werden können.	O	O	O	O	O	O	O
Es wäre mir unangenehm, weitere Informationen von mir abzugeben, (auch) wenn es um Entscheidungen bezüglich des gesellschaftlichen Lebens geht.	O	O	O	O	O	O	O
Ich würde mir keine Sorgen machen, wenn ich mehr Daten abgebe, die weitere Entscheidungen ermöglichen	O	O	O	O	O	O	O
Es wäre für mich in Ordnung, weitere Daten abzugeben, die als Basis für Maßnahmen genutzt werden könnten.	O	O	O	O	O	O	O
Ich würde mich beobachtet fühlen, wenn ich meine Daten abgebe, unabhängig davon, wofür diese genutzt werden.	O	O	O	O	O	O	O
Es würde mich beunruhigen, weitere Daten von mir abzugeben, die für Entscheidungen genutzt werden.	O	O	O	O	O	O	O
Es würde meine Privatsphäre einschränken, meine Daten abzugeben, (selbst) um diese für Maßnahmen einzubeziehen.	O	O	O	O	O	O	O
Ich fände es in Ordnung, wenn für eine solche Datenbasis weitere Informationen von mir gespeichert werden.	O	O	O	O	O	O	O

exploratory: perceived identity entrepreneurship by the government

Wie sehr stimmen Sie jetzt gerade spontan folgenden Aussagen zu?

Die Kommunikation der Regierung vermittelt mir...

	1 - trifft gar nicht zu	2	3	4	5	6	7 - trifft voll zu
dass ich Teil der Gesellschaft bin.	○	○	○	○	○	○	○
dass meine Handlungen bedeutungsvoll für das Wohlergehen aller sind.	○	○	○	○	○	○	○
dass sie an den richtigen Werten orientiert ist.	○	○	○	○	○	○	○
dass ich mit anderen Menschen verbunden bin.	○	○	○	○	○	○	○

Trust in the government

Bitte geben Sie an, wie Sie die Regierung in Bezug auf das Einsetzen dieser App und deren Daten einschätzen würden.

1. Wie vertrauenswürdig schätzen Sie die Regierung ein?
2. Wie ehrlich schätzen Sie die Regierung ein?
3. Wie kompetent schätzen Sie die Regierung ein?
4. Wie glaubwürdig schätzen Sie die Regierung ein?

Scale:

○ 1 - gar nicht

○ 2

○ 3

○ 4

◯ 5

◯ 6

◯ 7 - sehr

Having already installed the official Corona

Haben Sie die offizielle Corona-Warn-App bereits auf Ihrem Smartphone installiert?

◯ ja

◯ nein

◯ not sure / using a different app

Social Identification

1. Wie viel haben Sie Ihrer Meinung nach mit den folgenden Gruppen gemeinsam?
2. Wie stark fühlen Sie sich den folgenden Gruppen zugehörig?
3. Wie sehr fühlen Sie sich mit den folgenden Gruppen verbunden?
4. Wie viel Solidarität empfinden Sie mit den folgenden Gruppen?
5. Wie sehr identifizieren Sie sich mit der jeweiligen Gruppe (d.h. fühlen sich zugehörig zu ihr, empfinden Zuneigung, sorgen sich um diese)?
6. Wie betroffen fühlen Sie sich, wenn einer der folgenden Gruppen etwas Schlimmes passiert?

Scale:

	1 - gar nichts /keine	2	3	4	5	6	7 – sehr viel/ sehr stark
Mitglieder der Regierung	○	○	○	○	○	○	○
Menschen in meinem persönlichen Umfeld	○	○	○	○	○	○	○
Menschen auf der ganzen Welt	○	○	○	○	○	○	○

Exploratory: threat due to Corona / extent to which their personal lives have changed

Bitte beantworten Sie abschließend einige Fragen zu den Auswirkungen von Corona auf Ihren Alltag.

Wie besorgt fühlen Sie sich aktuell aufgrund der Corona-Situation?
Wie bedroht fühlen Sie sich aktuell aufgrund der Corona-Situation?
Wie sehr hat sich Ihr aktueller persönlicher Alltag (Freizeit) durch Corona verändert?
Wie sehr hat sich Ihr aktueller Studienalltag durch Corona verändert?

- ○ 1 - gar nicht
- ○ 2
- ○ 3
- ○ 4
- ○ 5
- ○ 6
- ○ 7 - sehr

Was denken Sie, wird in der Studie untersucht? (optional)

____

____

____

____

____

Demographics:

- Student / employed
- age
- gender
- tongue
- years having lived in Germany

Debriefing and thank you
